# Effectiveness and Mechanisms of Low-Intensity Pulsed Ultrasound on Osseointegration of Dental Implants and Biological Functions of Bone Marrow Mesenchymal Stem Cells

**DOI:** 10.1155/2022/7397335

**Published:** 2022-09-26

**Authors:** Chao Liang, Xiu Liu, Yuwei Yan, Rongxin Sun, Jun Li, Wei Geng

**Affiliations:** ^1^Department of Dental Implant Center, Beijing Stomatological Hospital, School of Stomatology, Capital Medical University, Beijing 100050, China; ^2^Beijing Institute of Dental Research, Beijing Stomatological Hospital, School of Stomatology, Capital Medical University, Beijing 100050, China

## Abstract

Dental implant restoration is the preferred choice for patients with dentition defects or edentulous patients, and obtaining stable osseointegration is the determining factor for successful implant healing. The risk of implant failure during the healing stage is still an urgent problem in clinical practice due to differences in bone quality at different implant sites and the impact of some systemic diseases on bone tissue metabolism. Low-intensity pulsed ultrasound (LIPUS) is a noninvasive physical intervention method widely recognized in the treatment of bone fracture and joint damage repair. Moreover, many studies indicated that LIPUS could effectively promote the osseointegration of dental implants and improve the osteogenic differentiation of bone marrow mesenchymal stem cells (BMSCs). This review is aimed at investigating the research progress on the use of LIPUS in dental implant medicine from three aspects: (1) discuss the promoting effects of LIPUS on osseointegration and peri-implant bone regeneration, (2) summarize the effects and associated mechanisms of LIPUS on the biological functions of BMSCs, and (3) introduce the application and prospects of LIPUS in the clinical work of dental implantation. Although many challenges need to be overcome in the future, LIPUS is bound to be an efficient and convenient therapeutic method to improve the dental implantation success rate and expand clinical implant indications.

## 1. Introduction

With the advancement of dental implant science, implant restoration has become the preferred treatment approach for patients with dentition defects or edentulous to restore oral function and aesthetics [1]. Osseointegration is an important metabolic and remodeling process involving bone tissues surrounding implant surfaces, and achieving stable osseointegration during the healing period is a prerequisite for successful dental implantation [2]. The theory of implant osseointegration was first proposed by Brånemark in 1977, who reported a direct structural and functional connection between the surface of a titanium implant and the active human bone tissues, without any connective tissue between these two components [3]. The quality of osseointegration is primarily influenced by the bone quality and bone mass at the local implant site, and systemic health factors that influence bone metabolism also play important roles [4].

Good bone quality and adequate bone mass can ensure that dental implants are placed at the ideal site, leading to a good functional and aesthetic outcome. However, periodontal disease, trauma, and bone tissue atrophy or resorption often cause alveolar bone insufficiency, and thus, different degrees of peri-implant bone defects appear after the implants are placed. Therefore, bone augmentation surgery, such as guided bone regeneration (GBR), is required to cover the exposed surface of the implant [5]. However, the period for achieving osseointegration in the bone defect region is even longer and is closely associated with the supply of peripheral blood and the migration and differentiation of osteoblastic cells in the bone marrow [6]. At present, firmly and quickly establishing osseointegration in the bone defect area around the implant is still a clinical challenge.

Furthermore, systemic diseases, such as diabetes mellitus and osteoporosis, are considered important risk factors that impact the success rate of dental implant treatment. These diseases are usually accompanied by different degrees of bone remodeling disorders, which can interfere with the osseointegration of implants during the healing period. Although diabetes mellitus with good blood glucose control and osteoporosis under systemic medication is no longer absolute contraindications for implant surgery because of the progress in implant surface treatment technology [7], several studies have reported that the aforementioned diseases still present a potentially high risk for implant failure [8, 9]. An absence of osseointegration directly causes implant loss and surgical failure and is difficult to predict. Therefore, finding new methods to improve the osseointegration of implants simply and quickly and shortening the healing cycle have become the focus of current clinical research.

Low-intensity pulsed ultrasound (LIPUS) is an emerging noninvasive technology for physical intervention and can directly act on target tissues using pulsed ultrasound at an output intensity lower than 1 W/cm^2^ to produce many biological effects, including promoting protein synthesis, improving cell proliferation, and increasing cellular secondary messenger calcium uptake [10, 11]. LIPUS is widely recognized as a safe and effective method for treating bone, cartilage, nerve, and soft tissue diseases and has almost no toxic or side effects to normal tissue [12–15]. Many studies have reported the promoting effect of LIPUS on tissue regeneration and cell metabolism, particularly in treating bone fracture and cartilage injury [16–18]. A systemic review and meta-analysis even defined LIPUS as the most effective method for treating bone nonunion besides surgery [19]. In addition, several studies explored the therapeutic effects of LIPUS on cartilage tissue injury of the temporomandibular joint (TMJ) in the last 5 years and found that LIPUS could effectively suppress temporomandibular joint disorders (TMDs) in rats, which was caused by chronic sleep deprivation (CSD) intervention [20–22]. A recent review article also confirmed the effect of LIPUS on osteoarthritis of the TMJ [23].

In recent years, many studies used LIPUS in the field of dental implantation, seeking to use LIPUS to improve peri-implant bone remodeling and shorten the healing cycle. *In vivo* studies showed that LIPUS could significantly increase the bone–implant contact (BIC) rate of implants and effectively promote new bone formation [24]. *In vitro* studies further confirmed that LIPUS could promote the proliferation, migration, osteogenic differentiation, and mineralization abilities of alveolar bone marrow mesenchymal stem cells (BMSCs), activate osteogenesis-associated signaling pathways, and induce BMSCs to express osteogenic cytokines and proteins [16]. LIPUS has high clinical application value in the promotion of implant osseointegration and bone regeneration around implants during the patient healing period. The present study investigated the research progress in LIPUS use in dental implant medicine from three aspects: (1) the promotive effects of LIPUS on implant osseointegration and peri-implant bone regeneration, (2) the effects and associated mechanisms of LIPUS on the biological functions of BMSCs, and (3) the application and prospects of LIPUS in the clinical work of dental implantation. Finally, future research directions have been suggested in Conclusion.

## 2. Promotive Effects of LIPUS on Implant Osseointegration and Peri-Implant Bone Regeneration

In the last 30 years, the treatment of bone fractures and other bone defect diseases with LIPUS has achieved landmark clinical effects. The osseointegration of implants shares many similarities with the bone fracture healing process, including blood clot filling, inflammatory response, osteoid tissue formation, and bone remodeling [25]. After implants are placed into the alveolar bone tissues, blood first fills the gap between the implant surface and the surrounding bone tissue, and then, osteoid tissues and new trabecular bone gradually replace the blood clots during the early healing stage. Next, the bone-like tissues are gradually remodeled to form lamellar bone to achieve close contact with the implant surface, and osseointegration is finally complete [26]. Many studies have verified the promoting effect of LIPUS in bone remodeling and regeneration, and in the following paragraphs, we will focus on the effects of LIPUS on implant osseointegration. Current *in vivo* studies on the application of LIPUS to dental implants are summarized in Table 1.

### 2.1. Interventional Effects of LIPUS on Implant Osseointegration

Many *in vivo* studies used LIPUS to promote the osseointegration of dental implants. Ustun et al. [27] showed that the intervention of dental implants in rabbit tibias for 4–6 weeks using LIPUS at 30 mW/cm^2^ intensity significantly increased the BIC rate and the stability of implants. Liu et al. [32] used 40 mW/cm^2^ LIPUS to treat implants in rabbit femurs and tibias, and 3 weeks of intervention significantly increased the tissue mineral density, bone volume/tissue volume (BV/TV) fraction, trabecular thickness around the implants, and pullout torque of the implants. Similarly, Zhou et al. [29] in a rat model showed that the application of LIPUS at 30 mW/cm^2^ significantly increased the BIC rate and the BV/TV fraction in rat tibias in week 4 compared with the natural healing control group. However, the differences were not significant in weeks 8 and 12, suggesting that the advantage period of LIPUS in promoting new bone formation around implants was during the early healing period. Simultaneously, Kang et al. [28] also concluded that LIPUS could effectively promote the osseointegration of dental implants in 4 weeks in a canine model. In addition, Ruppert et al. [34] compared the effects of LIPUS and low-magnitude, high-frequency (LMHF) vibration generated by a dual-limb local vibration stimulator on implant healing in rat femurs and showed that LIPUS promoted osseointegration after 4 weeks of intervention and increased the pullout torque of implants more significantly than the vibration stimulator, allowing implants to achieve the stable plateau stage earlier. However, the aforementioned promoting effects disappeared after 8 weeks. In summary, the aforementioned studies showed that LIPUS strongly and stably promoted implant osseointegration and peri-implant bone regeneration, and the promoting function primarily occurred in the early period of osseointegration (about 4 weeks).

**Figure 1:**
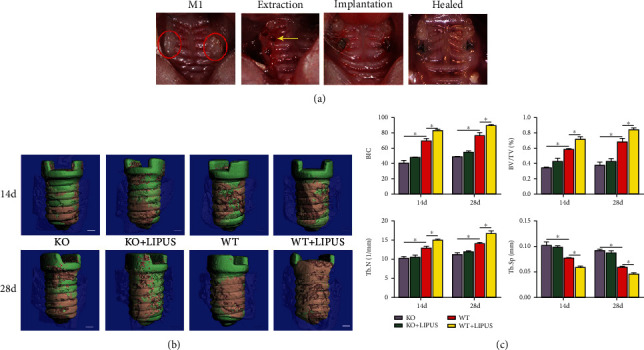
LIPUS enhanced the osseointegration of dental implant in *α*CGRP^+/+^ mice. (a) Tooth extraction and implant placement procedure. The red circles indicate bilateral maxillary first molars. The yellow arrow points to the palatal root socket after tooth extraction. (b) Three-dimensional reconstruction of the implant by microcomputed tomography (micro-CT). The green area indicates implant, and the pink area indicates BIC. Scale bars = 100 mm. (c) Micro-CT analysis of BIC, BV/TV, Tb.N, and Tb.Sp. Data are presented as means ± standard deviation. ^∗^*P* < 0.05, *n* = 4 specimens/group. KO: *α*CGRP knockout mice; WT: wild type; BIC: bone–implant contact; BV/TV: bone volume/tissue volume fraction; Tb.N: mean trabecular number; Tb.Sp: mean trabecular separation. Reprinted from Jiang et al. [24], Copyright (2020), with permission from Elsevier.

In a recent study, Jiang et al. [24] also confirmed the promoting effects of LIPUS at 30 mW/cm^2^ intensity on implant osseointegration in 4 weeks, and the aforementioned function was produced through the promotion of *α*-calcitonin gene-related peptide (*α*CGRP) synthesis and secretion by dorsal root ganglia neurons. CGRP is a neuropeptide that regulates the biological activities of nonneural cells, and the major function of *α*CGRP is to regulate bone formation and remodeling [35]. Jiang et al. [24] showed that LIPUS intervention significantly promoted BIC, BV/TV, and the mean trabecular number (Tb.N) and decreased the mean trabecular separation (Tb.Sp). However, LIPUS did not have significant effects in *α*CGRP knockout mice (Figure 1). Therefore, *α*CGRP might be a hub through which LIPUS promoted implant osseointegration, and this conclusion provided a new perspective for the exploration of the mechanism of action of LIPUS.

### 2.2. Effect of LIPUS on Implant Osseointegration in the Presence of Osteoporosis and Diabetes Mellitus

Osteoporosis is a common human bone tissue disease primarily characterized by reduced bone density [36]. The reduction in bone mass and volume caused by bone metabolism imbalances not only affects implant osseointegration during the healing period but is also an important risk factor for a decrease in long-term implant survival rates [37, 38]. Systemic bisphosphate, estrogen, or parathyroid hormone treatment can inhibit the activity of osteoclasts in osteoporotic bone tissues and enhance osseointegration [39, 40]. However, treatment with the aforementioned systemic drugs may lead to many adverse reactions and toxic drug effects, of which osteonecrosis of the jaw bone induced by bisphosphate drugs is the most serious [41]. In this respect, LIPUS has unique advantages because it is nontoxic, nonimmunogenic, and noninvasive. Zhou et al. [33] confirmed that LIPUS could effectively promote the osseointegration of titanium implants in osteoporotic bone tissues. Treatment with LIPUS at 40 mW/cm^2^ intensity for 2 weeks significantly increased the BV/TV fraction around implants in the femurs of ovariectomized rats; treatment for 4 weeks or more significantly increased the BIC rate of the implants; and treatment for 6 weeks or more significantly increased the pullout torque. In addition, the present study confirmed that the enrichment and osteogenic differentiation of osteoblasts close to the implant–bone interface stimulated by LIPUS was an important route through which LIPUS exerted its promoting effect on osseointegration. The aforementioned results provided a theoretical basis for the application of LIPUS to assist the healing of dental implantation in patients with osteoporosis.

Diabetes mellitus is another important risk factor for implant failure during the healing period [42–44]. Many studies confirmed that the BIC rate of implants in Goto-Kakizaki (GK) rats with type 2 diabetes mellitus was significantly lower than that in normal Wistar rats, and hyperglycemia played a key role in causing the bone remodeling disorders around the implants [45–47]. In addition, cellular studies showed that a high-glucose microenvironment could significantly inhibit the proliferation and osteogenic differentiation capacity of BMSCs, reduce the expression of osteogenesis-related genes, and decelerate *in vitro* mineralization [48–50]. Unfortunately, to the best of our knowledge, currently, no relevant clinical or *in vivo* studies have reported the function and mechanism of LIPUS in the osseointegration of implants in diabetic models. However, in the field of bone fracture treatment, LIPUS significantly promoted bone healing and angiogenesis in rats with diabetes and increased the healing speed to a degree similar to that observed in normal rats [51, 52]. Based on these studies and the studies of LIPUS in the promotion of bone regeneration around implants, we speculated that LIPUS could also be used as an effective adjunct treatment method to improve implant osseointegration in patients with diabetes. However, future *in vivo* studies and clinical studies are still necessary for confirmation and more in-depth exploration.

### 2.3. Optimal LIPUS Treatment Parameters for Implant Osseointegration

In current studies, the parameters of LIPUS applied to dental implants, such as intensity, frequency, and intervention cycle, primarily referred to previous studies on bone fracture treatment. Since Duarte [53] used LIPUS for promoting bone fracture healing in 1983, studies on LIPUS functions mostly adopted a stimulation duration of 20 min/day. The selection of this duration not only effectively promoted bone regeneration and shortened the healing cycle but also avoided the physical and mental fatigue of patients caused by overly long intervention times. In addition, in currently published studies, the ultrasound intensity used to promote implant osseointegration and peri-implant bone regeneration was typically 30–40 mW/cm^2^, and this intensity achieved good therapeutic effects [24, 27, 29, 32–34]. Nakanishi et al. [30] showed that the promoting effect of LIPUS at 40 mW/cm^2^ intensity on the osseointegration and stability of implants in rabbit femurs was higher than that at 100 mW/cm^2^ intensity. Similarly, cell experiments showed that LIPUS at 40 mW/cm^2^ intensity induced more significant *in vitro* mineralization of the mouse osteoblast cell line MC3T3-E1 compared with 120 mW/cm^2^ intensity [54]. Therefore, it was speculated that the vibration and heat generated by higher LIPUS intensities might have negative effects on the biological functions of bone-derived cells, lowering the efficacy of LIPUS compared with that at 30–40 mW/cm^2^ intensity. For the ultrasound frequency, studies mainly used a fixed frequency of 1.5 MHz due to the limitation of the LIPUS instrument [27, 29, 32–34]. Only Nakanishi et al. [30] compared the effects of LIPUS on osseointegration at different frequencies, and showed that the promoting effect of LIPUS at a frequency of 3 MHz was higher than that at 1 MHz. In addition, Hsu et al. [31] applied pulsed-wave and continuous-wave ultrasound to treat implants in rabbit tibias for 30 days and showed that new bone formation around implants in the pulsed-wave groups was faster and observed more mature type I collagen expression and angiogenesis around the implants. The present study confirmed that the effect of LIPUS on implant osseointegration was better than that of low-intensity continuous ultrasound (LICUS).

**Figure 2:**
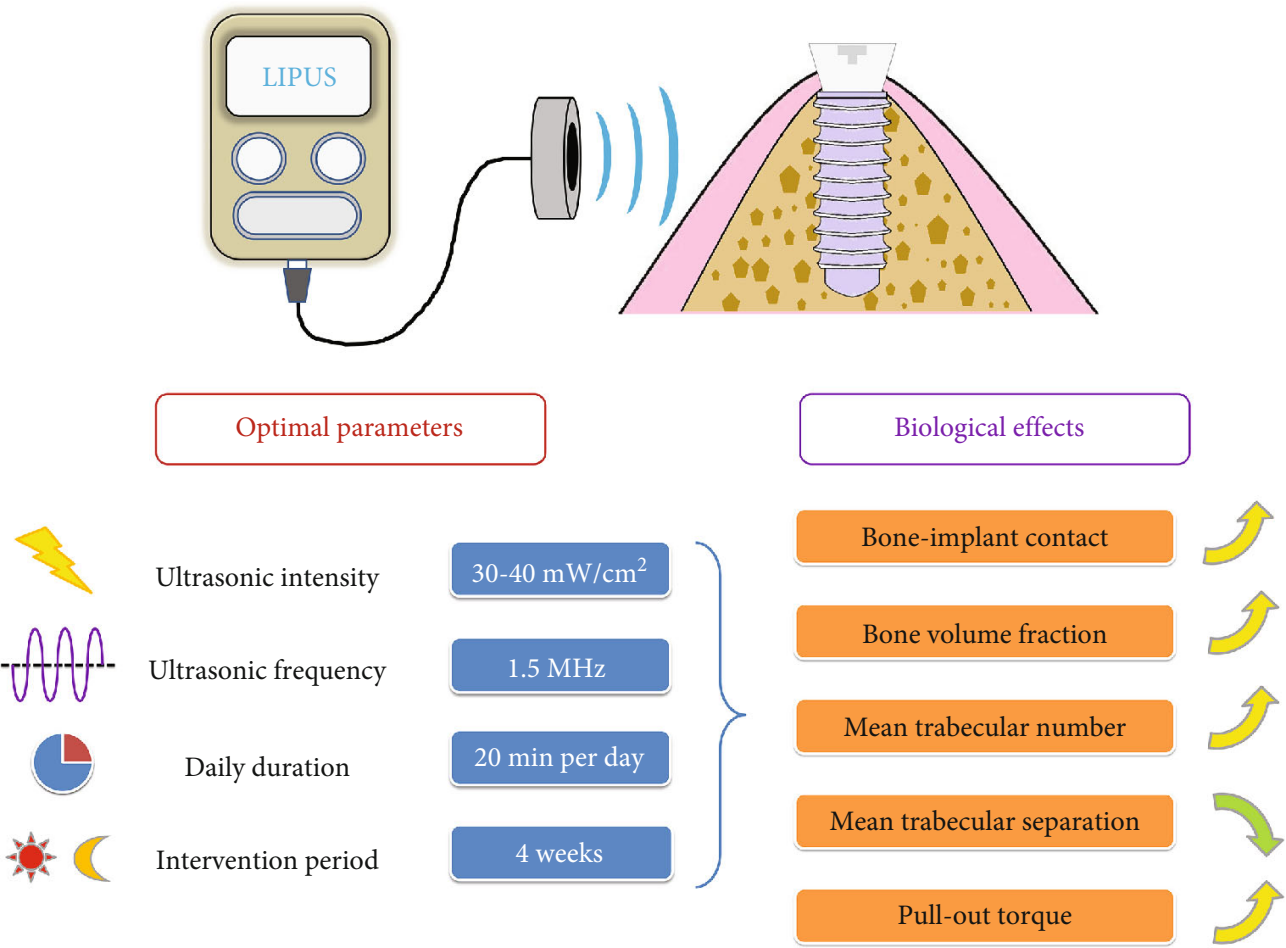
Schematic diagram of the optimal parameters and biological effects of LIPUS used in dental implantations. Based on the results of previous studies, LIPUS intervention at 30–40 mW/cm^2^ intensity and 1.5 MHz frequency for 20 min/day for 4 weeks is the most commonly used and effective scheme for promoting dental implant osseointegration.

The results of the aforementioned studies could be summarized as follows: LIPUS intervention at an intensity no higher than 50 mW/cm^2^ (recommendation: 30–40 mW/cm^2^) and a frequency of 1.5 MHz for 20 min/day for 4 weeks is currently the most commonly used and effective scheme for promoting dental implant osseointegration (Figure 2). However, this point of view still requires to be confirmed by controlled *in vivo* experiments with strict grouping in the future.

## 3. Effects and Associated Mechanisms of LIPUS on the Biological Functions of BMSCs

The osteogenic differentiation and bone-forming functions of bone-derived cells play key roles throughout the entire implant osseointegration process. In the early stage, after implants are installed into the jaw bone, extracellular matrix (ECM) proteins in the blood are rapidly adsorbed to the surface of implants to form a “protein layer” [55, 56]. By recognizing the Arg-Gly-Asp tripeptide sequence (RGD sequence) on the “protein layer,” BMSCs and other osteogenic precursor cells begin to anchor on the surface of the implants [57], initiating subsequent proliferation and differentiation processes and synthesizing osteogenesis-associated proteins [58, 59]. Therefore, the adhesion, proliferation, and differentiation of bone-derived cells on the surface of the implant were the initial steps in the early stage of osseointegration [60].

Previous studies showed that LIPUS could be used as an effective external stimulus to improve bone regeneration around biomaterials through the promotion of proliferation and differentiation of BMSCs and osteoblasts. Moonga et al. [61] showed that LIPUS enhanced matrix mineralization of mouse MC3T3-E1 osteoblasts in bovine trabecular bone scaffold materials. Carina et al. [62] showed that LIPUS significantly promoted the osteogenic differentiation of human mesenchymal stem cells (MSCs) cultured in a mixed Mg-hydroxyapatite/collagen scaffold material. Zhou et al. [63] found that LIPUS treatment enhanced the proliferation ability of human BMSCs on 3D-bioprinted tissue scaffolds and increased the expression of alkaline phosphatase (ALP) and matrix mineralization. An et al. [64] showed that LIPUS significantly promoted the adhesion and proliferation of rat BMSCs on the surface of titanium implants, and the osteogenesis-related genes osteopontin (OPN), osteocalcin (OCN), bone morphogenetic protein-2 (BMP-2), ALP, Runt-related transcription factor 2 (Runx2), and collagen type I were upregulated under LIPUS stimulation to improve implant osseointegration.

**Figure 3:**
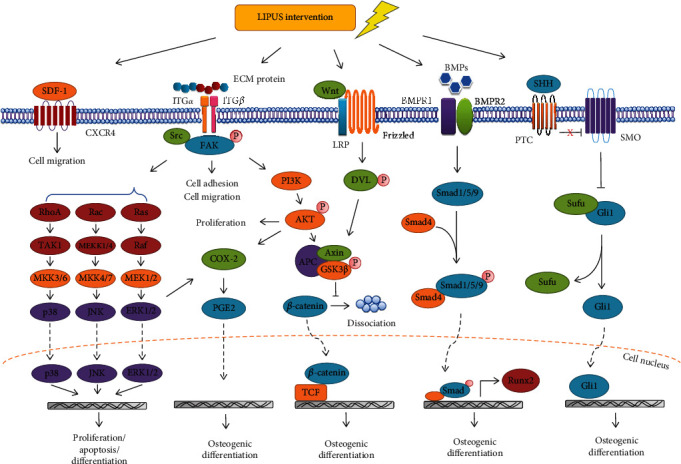
Schematic diagram of signaling pathways that can be activated by LIPUS in bone-derived cells for regulating cell biology functions and implant osseointegration, which include integrin and focal adhesion pathway, MAPK pathway, SHH pathway, BMP/Smad pathway, SDF-1/CXCR4 signaling, and COX-2/PGE2 signaling.

The mechanisms through which LIPUS exerts its promoting effects on cell metabolism and tissue repair are complex and still not fully understood, but it is generally recognized that they might be associated with the mechanical stress and/or fluid microstreaming effect of LIPUS [65–68]. Ultrasonic waves can produce a weak oscillatory force resulting in potential changes in body tissues; such forces can act on the ECM, transmembrane proteins, and intracellular fluids to convert mechanical signals into biochemical signals that affect target gene expression and cellular functions [23, 69]. The already known signal transduction pathways regulated by LIPUS mainly include the integrin and focal adhesion signaling pathway, mitogen-activated protein kinase (MAPK) signaling pathway, sonic hedgehog (SHH) signaling pathway, BMP/Smad signaling pathway, cyclooxygenase-2 (COX-2)/prostaglandin E2 (PGE2) signaling, and stromal cell–derived factor-1 (SDF-1)/C-X-C chemokine receptor type 4 (CXCR4) signaling. These signaling pathways can eventually activate bone-derived cell adhesion, migration, proliferation, and osteogenic differentiation to stimulate new bone formation and promote implant osseointegration. The mechanistic studies of these pathways can elucidate the phenomena observed during *in vivo* studies from different perspectives. The aforementioned pathways associated with the regulation of bone-derived cell biology functions and implant osseointegration promoted by LIPUS are summarized in Figure 3.

### 3.1. Integrin and Focal Adhesion Signaling Pathway

Integrins are a family of transmembrane proteins that mediate the connection between cells and the extracellular environment. They are also important mechanoreceptors of cells that convert the mechanical signals of LIPUS into biochemical signals. Many studies showed that LIPUS stimulation could regulate the expression level of integrin on the cell membrane. Chen et al. [70] showed that LIPUS at 60 mW/cm^2^ intensity significantly increased integrin alpha 8 (ITGA8) expression in rat BMSCs and promoted the migration ability of the cells through the focal adhesion signaling pathway. Xiao et al. [71] found that after LIPUS intervention, the expression of integrin *β*1 in rat BMSCs increased and the cell migration ability significantly enhanced. The “ECM–integrin–focal adhesion–cytoskeleton” connection is the main pathway involved in the transmission of the LIPUS signal into cells to exert biological effects [72]. Focal adhesions are large protein complexes that connect ECM proteins and intracellular cytoskeletal proteins and are hubs for regulating cell adhesion, migration, and signal transduction [73]. When LIPUS signals are transmitted to integrin, focal adhesion kinase (FAK) is first phosphorylated to initiate the focal adhesion signaling pathway [74, 75], and phosphorylated FAK can activate the downstream PI3K/Akt signaling pathway to regulate the proliferation and differentiation of osteogenesis-associated cells [76].

Tang et al. [77] showed that the treatment of rat primary osteoblasts with LIPUS upregulated the expression of integrins *α*2, *α*5, *β*1, and *β*3 on the cell membrane and promoted osteoblast differentiation and bone formation through the ITG/FAK/PI3K/Akt signaling pathway. Xie et al. [78] showed that LIPUS treatment at 50 or 60 mW/cm^2^ intensity for 5 min/day effectively promoted the proliferation ability of human BMSCs by activating the PI3K/Akt signaling pathway. Watabe et al. [79] found that LIPUS stimulation significantly upregulated integrin *α*5 (ITGA5) gene expression in mouse osteoblasts derived from the long bone, mandible, and cranial parietal bone and promoted the expression of the osteogenesis-related genes ALP and Runx2 by activating the PI3K/Akt signaling pathway.

In addition, studies found that LIPUS could also activate *β*-catenin signaling to significantly influence osteoblast differentiation and bone tissue regeneration [80]. Akt activation could further induce the phosphorylation of glycogen synthase kinase 3 beta (GSK3*β*), inactivate the APC–Axin–GSK3*β* complex, and inhibit the dissociation of *β*-catenin, causing *β*-catenin to accumulate and enter the nucleus to promote the transcription and synthesis of osteogenesis-associated factors [81, 82]. Thus, it was speculated that LIPUS stimulation could activate classical Wnt/*β*-catenin signaling through the focal adhesion signaling pathway, thus promoting new bone formation and implant osseointegration. However, the aforementioned mechanism still requires further studies for confirmation.

### 3.2. MAPK Signaling Pathway

MAPKs can be activated by cell stress responses induced by extracellular mechanical stimulation, mediating the transduction of mechanical signals to regulate cell proliferation and differentiation [83]. The MAPK pathway also plays an important role in the biological processes of osteoblast differentiation and bone formation [84].

The ITG/FAK/MAPK signaling pathway is a canonical pathway regulating the biological activity of bone-derived cells. FAK phosphorylation induced by LIPUS stimulation further activates three important components, extracellular signal–regulated kinase (ERK), Jun N-terminal kinase (JNK), and p38, of the downstream MAPK pathway [85]. ERK signaling is primarily activated through the Ras/Raf/MEK/ERK pathway to regulate cell proliferation, migration, differentiation, aging, and apoptosis [86]. Current studies generally considered that Runx2 phosphorylation could be activated by ERK signaling, which was an important mechanism underlying the promotion of osteogenic differentiation [87]. In addition, JNK activation plays a key role in cell proliferation, apoptosis, and differentiation. However, the effect of JNK on osteogenic differentiation is controversial. Some studies showed that JNK activation could inhibit the adipogenic differentiation of stem cells and promote osteogenic differentiation [88]. Other studies showed that the inhibition of JNK phosphorylation in stem cells increased the ALP expression level and promoted the osteogenic differentiation ability of the cells [89]. p38 MAPK is a stress-activated protein kinase (SAPK) that can be activated by endogenous and exogenous stimuli through MAP kinase kinase (MKK) 3/6 to participate in the stress responses of cells and regulate cell proliferation, apoptosis, and chromatin remodeling [90]. In addition, p38 activation is necessary for osteoblast differentiation [91, 92], can be activated by BMP signaling, and synergistically promotes osteoblast differentiation with Smad signaling [93, 94].

Gao et al. [95] found that LIPUS could regulate the proliferation and apoptosis of different dental stem cell populations through the MAPK signaling pathway. JNK signaling was activated by LIPUS in BMSCs, and specific inhibition of the JNK pathway blocked the promoting effect of LIPUS on cell proliferation. Kaur et al. [96] showed that LIPUS stimulated ERK1/2 activation in MC3T3-E1 mouse osteoblasts and upregulated the expression of Runx2, OCN, and OPN genes. Angle et al. [97] showed that LIPUS at 2, 15, or 30 mW/cm^2^ intensities regulated the activation of ERK1/2 and p38 in rat BMSCs, thus regulating cell osteogenic differentiation. In addition, Kusuyama et al. [98] showed that LIPUS promoted the expression of Cot/Tpl2 kinase in MSCs and further regulated MEK1 and ERK phosphorylation to inhibit adipogenic differentiation and promote the osteogenic differentiation of the cells. In summary, as a group of important signaling molecules downstream of FAK, MAPK pathway members played important roles in the transition of MSCs into osteoblast cell lines under LIPUS stimulation.

### 3.3. SHH Signaling Pathway

The SHH signaling pathway is a classical pathway that regulates body development and homeostasis and plays an important role in bone remodeling and regeneration [99, 100]. After bone-derived cells are subjected to external stimulation, SHH in the ECM begins to interact with its membrane receptor Patched (Ptc) to relieve the inhibition on Smoothened (Smo) protein, subsequently promoting the entry of Gli protein into the nucleus to further activate the transcription of downstream osteogenesis-associated target genes [101], which directly affect the transformation of MSCs into osteoblast cell lines [102]. Zhou et al. [103] showed that LIPUS promoted the migration and proliferation of MG63 osteoblast-like cells to accelerate bone formation and the SHH inhibitor GDC0449 significantly inhibited the aforementioned functions of LIPUS. Matsumoto et al. [104] found that LIPUS significantly increased the expression of the functional genes Gli1 and Gli2 in the SHH signaling pathway and promoted the osteogenic differentiation of MC3T3-E1 cells and accelerate bone tissue regeneration by activating the SHH pathway. In addition, another study showed that activated SHH signaling could promote the osteogenesis-related gene expression of MC3T3-E1 cells by upregulating FAK phosphorylation at Tyr397 [105]. Therefore, LIPUS stimulation could not only activate the SHH signaling pathway but also interact with FAK-associated pathways to promote the osteogenic differentiation of osteoprogenitor cells and bone remodeling.

### 3.4. BMP/Smad Signaling Pathway

BMPs are a group of secretory proteins in the transforming growth factor-*β* superfamily that play critical roles in the regulation of bone metabolism [106]. After interaction with type I and type II transmembrane serine/threonine kinase receptors (BMPR-I and BMPR-II) on the cell surface, BMPs can transduce external stimulus signals into cells to regulate the osteogenic differentiation of BMSCs [107, 108]. BMP-2 is a classic osteogenesis-promoting protein. Many studies found that LIPUS could significantly promote BMP-2 synthesis and secretion in bone-derived cells to improve bone metabolism and promote bone formation [64, 109–111].

BMP-2 signaling is transmitted by intracellular signal transduction proteins called Smads. When BMP-2 interacts with its membrane receptors, Smads 1/5/9 begin to be phosphorylated and activated. Then, p-Smad 1/5/9 and Smad 4 oligomerize to form a complex and are transported into the nucleus to regulate the expression of downstream genes. Synthesis of Runx2 and many other bone formation-related factors could be stimulated by the activated BMP-2/Smad signaling pathway [106]. Maung et al. [112] showed that LIPUS significantly increased the expression of BMP-2 in periosteal cells and promoted Smad1/5/9 phosphorylation, thus enhancing the transcription of osterix (OSX) and improving the osteogenic differentiation potential of these cells. Zhang et al. [113] showed that LIPUS at 20 or 30 mW/cm^2^ intensity effectively promoted BMP-2 and BMP-7 expression in stem cells, thus stimulating the osteogenic differentiation of the cells and inducing Runx2, OCN, and OPN expression by promoting Smad1/5 phosphorylation. Runx2 is a transcription factor with an important role in the bone formation process. Studies found that the expression of Runx2 in rat osteoblasts and BMSCs was significantly upregulated after LIPUS stimulation to promote the osteogenic differentiation of the cells [114–116]. Therefore, as a canonical regulatory pathway for osteogenic differentiation, the BMP/Smad/Runx2 pathway activated by the ultrasonic wave in various bone-derived cells is also an important mechanism for LIPUS to exert its biological functions.

### 3.5. SDF-1/CXCR4 Signaling

In the early stage of bone tissue repair or implant osseointegration, BMSCs can be recruited to the injured regions or implant sites to exert biological functions, and in this process, cell migration and chemotaxis play important roles. SDF-1 and its specific receptor, CXCR4, are key factors that regulate the migration of BMSCs to the bone remodeling site for promoting bone fracture repair, distraction osteogenesis, extraction socket healing, and implant osseointegration [117–119]. Wang et al. [120] confirmed that LIPUS could stimulate SDF-1 secretion in stem cells and promote cell migration ability through the SDF-1/CXCR4 pathway. Xiao et al. [71] showed that LIPUS significantly promoted the migration and chemotaxis of rat BMSCs, upregulated SDF-1 and CXCR4 mRNA expression in the cells, and increased SDF-1 protein synthesis and secretion. However, after cell treatment with the SDF-1/CXCR4 pathway inhibitor AMD3100, the aforementioned functions of LIPUS were almost completely blocked. Wei et al. [121] showed that LIPUS promoted rat BMSC migration to bone tissue repair areas and observed that SDF-1 expression was upregulated in the local repair areas and the serum. Similarly, after the inhibition of SDF-1/CXCR4 signaling, the stimulatory function of LIPUS was significantly reduced. The promotion of SDF-1-mediated BMSC migration by LIPUS is also a key mechanism underlying the stimulation of implant healing because the implant osseointegration process is the process of bone tissue repair and regeneration around the implants.

### 3.6. COX-2/PGE2 Signaling

PGE2, a metabolite derived from arachidonic acid, has been shown to be upregulated when bone-derived cells perform their biological functions, and the expression of PGE2 is closely associated with bone remodeling and regeneration [122]. Studies showed that LIPUS could effectively increase COX-2 gene expression in osteoblasts and thereby promote PGE2 expression [123, 124]. Kokubu et al. [68] verified that COX-2 was the rate-limiting enzyme in PGE2 synthesis during LIPUS stimulation in MC3T3-E1 cells. Pretreatment of cells with specific COX-2 inhibitors could block the promoting effect of LIPUS on PGE2 expression and weaken the osteogenic ability of the cells. Naruse et al. [125] showed that the speed of bone remodeling significantly decreased in COX-2 gene knockout mice. In addition, the promotion of bone regeneration induced by LIPUS stimulation was also significantly inhibited. However, the injection of PGE2 receptor agonists restored the sensitivity of mice to LIPUS intervention. Furthermore, Hidaka et al. [126] found that LIPUS intervention increased the PGE2 level in the microenvironment of bone tissue repair areas and recruited BMSCs through PGE2 to promote local bone regeneration. Tang et al. [77] further confirmed that LIPUS stimulated COX-2 expression through the FAK/PI3K/Akt and ERK1/2 signaling pathways in MC3T3-E1 cells and upregulated PGE2 synthesis, which effectively promoted osteoblast differentiation and bone formation. Therefore, PGE2 could be directly regulated by COX-2 and might be the key target of LIPUS stimulation for bone regeneration and implant osseointegration.

Thus far, although many studies have elucidated the signaling pathways through which LIPUS regulates osteogenesis-associated cellular functions and promotes new bone formation, how these pathways interact and which pathway plays the most important role during implant healing are still not clear. In the future, *in vivo* implant models should be used for more intuitive validation and further investigation of the mechanism of action.

## 4. Application and Prospects of LIPUS in the Clinical Work of Dental Implantation

In recent years, LIPUS has been widely used as a convenient and effective method to promote fracture healing and bone defect repair. LIPUS was approved by the US Food and Drug Administration (FDA) as early as in 1994 and 2000 for accelerating fresh fracture healing and reconstitution of bone nonunion [127]. At present, the clinical application of LIPUS in dental implantation is still in its infancy. However, according to the existing *in vivo* studies and cell biology studies, we speculate that LIPUS may have good application value in promoting the osseointegration of implants in the future clinic practice.

**Figure 4:**
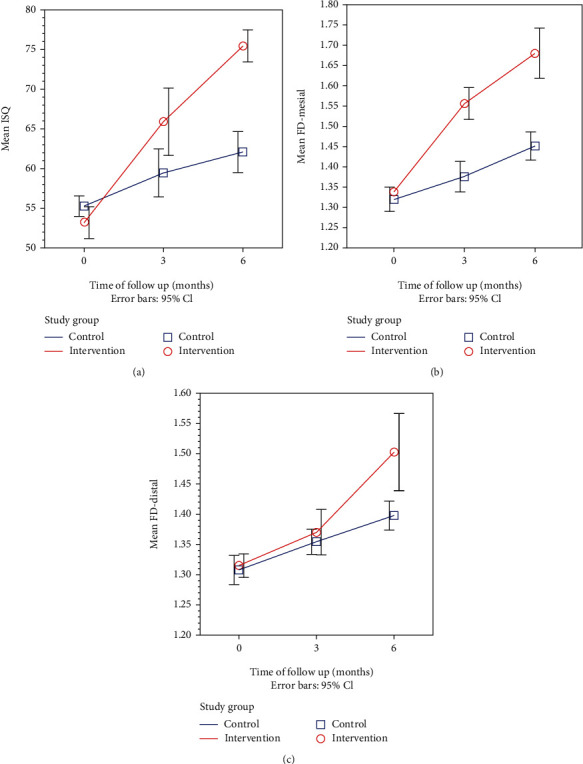
LIPUS stimulation could significantly improve implant stability assessed by both resonance frequency (RF) analysis and fractal dimension (FD) analysis. Line graphs showing the time trend for (a) mean RF values, (b) mesial side mean FD values, and (c) distal side mean FD values after surgery in the intervention group compared with the control group. Adapted from Abdulhameed et al. [129].

In a clinical study by Abdulhameed et al. [128], LIPUS was applied to patients with dental implants in the premolar region to accelerate osseointegration. After 2 weeks of the implantation surgery, LIPUS intervention at 30 mW/cm^2^ intensity and 1.5 MHz frequency was used for 10 weeks, with treatments twice a week for 20 min each time. Six months after the surgery, the clinical and imageological examinations showed that the marginal bone loss of the implants was lower in the LIPUS treatment group, vertical bone regeneration was observed, and the implant stability coefficient by resonance frequency (RF) analysis significantly increased compared with that in the conventional healing group. In another double-blind clinical study, this research group also confirmed that LIPUS stimulation could significantly improve implant stability assessed by both bone texture fractal dimension (FD) analysis and RF analysis (Figure 4) [129]. Thus, LIPUS could effectively promote implant osseointegration during the healing period and shorten the healing cycle in clinical patients. Furthermore, the aforementioned studies speculated that LIPUS could be used to save initially unstable implants and assist in obtaining higher-quality osseointegration, thus improving the success rate of implantation, especially in patients with osteoporosis and diabetes, which can affect bone remodeling.

In addition, Abdulhameed et al. observed an increase in the thickness of the buccal bone plate in the implantation area stimulated by LIPUS, with a statistically significant difference compared with the control group of patients who underwent conventional healing [128]. Moreover, Kim et al. [130] found that the local intervention with LIPUS in patients with maxillary sinus floor lift could effectively promote new bone formation, thus providing sufficient bone mass for the implant surgery in the maxillary posterior tooth area. Based on these studies, LIPUS may be used in patients undergoing bone augmentation surgery during or prior to implantation in the future to accelerate bone regeneration and shorten the treatment cycle of patients with insufficient alveolar bone for the implantation.

LIPUS has the advantages of low toxicity, low immunogenicity, noninvasiveness, high targeting selectivity, and repeatability [11, 23, 69]. The current clinical application of LIPUS has not caused any discomfort-related symptoms in patients, and no abnormal reactions, such as redness, swelling, or inflammation, have been observed in local soft tissues after the intervention. In addition, the portable LIPUS instrument is small in size and powered by a mobile unit, and the application is not limited by space. Therefore, in the future, as an effective, safe, and comfortable physical treatment method, LIPUS may lead to the adoption of a pattern of chair-side or household treatments to assist dentists and patients in achieving higher-quality implant osseointegration, promote bone regeneration in the defect area around the implant, and even prevent marginal bone loss and improve the long-term retention rate of the implant. However, more prospective cohort studies and randomized controlled trials (RCTs) are necessary in the future to confirm the function and mechanism of LIPUS and to determine the indications for LIPUS use in clinical practice of oral implantation, thus further supporting its application value and prospects.

## 5. Conclusions

As novel physiotherapy, LIPUS has been widely used in bone tissue, cartilage tissue, and soft tissue repair and reconstruction, and many studies have used it to promote the regeneration of oral and maxillofacial tissue. In the field of dental implantology, the application of LIPUS is still in its infancy. The existing studies provided a certain research foundation concerning the mechanism and clinical function of LIPUS, but further discussion is still needed.

In this review, based on the existing studies, it was found that LIPUS had an apparent promoting effect on dental implant osseointegration, suggesting that LIPUS could shorten the healing cycle after implant surgery and accelerate peri-implant bone reconstruction. Bone-derived cell adhesion, proliferation, migration, and differentiation on the surface of implants play a key role in the osseointegration process. This review systematically summarized the current role of LIPUS in the biological functions of the cells and related mechanisms. In addition, this review also addressed the application prospects of LIPUS in clinical dental implantation. Despite facing many challenges, based on the experience of LIPUS application in the treatment of bone tissue diseases, such as fracture and bone defect, the potential value of LIPUS in clinical dental implantation may be far beyond the existing reports.

Based on the in-depth exploration of the mechanism of LIPUS *in vitro*, we suggested that a transformation from a small-animal model to a large-animal model be considered for *in vivo* validation experiments. A model of jaw bone implantation with weight-bearing stress can be established, and the observation period can be further extended to more convincingly detect the effect of LIPUS. In addition, we also suggest exploring the therapeutic effect of LIPUS on the osseointegration of implants in abnormal microenvironments, such as in diabetes or osteoporosis, and clarifying the intervention effect of LIPUS on implants with poor initial stability or a poor healing state, so as to provide a new theoretical basis for improving the success rate of dental implantation and expanding the clinical indications.

## Figures and Tables

**Table 1:** Summary of LIPUS studies on implant osseointegration and peri-implant bone regeneration.

Studies	Animal models	Titanium implants	LIPUS parameters	Time of stimulation	Major conclusions
Ustun et al. [27]	New Zealand rabbits Tibiae	Length: 6.0 mm Diameter: 4.1 mm Screw-shaped	Intensity: 30 mW/cm^2^ (/SATA) Pulse frequency: 1.5 MHz	20 min/day for 1, 2, 3, 4, 5, and 6 weeks	LIPUS may have positive effects on osseointegration and stability of dental implants
Kang et al. [28]	Mongrel dogs Mandibular bone	Length: 8.5 mm Diameter: 3.3 mm	Intensity: 240 mW/cm^2^ (/SATA) Pulse frequency: 3.0 MHz	15 min/day for 1 week	LIPUS may have a positive effect on osseointegration and stability of dental implants, especially in early healing periods
Zhou et al. [29]	SD rats Tibiae	Length: 4.0 mm Diameter: 2.0 mm Pitch: 0.6 mm Screw-shaped	Intensity: 30 mW/cm^2^ (/SATA) Pulse frequency: 1.5 MHz	20 min/day for 4, 8, and 12 weeks	LIPUS therapy may accelerate the bone healing and osseointegration at the interlace between titanium implant and bone and promote remodeling of bone trabecula in the early stage
Nakanishi et al. [30]	Japanese white rabbits Femur	Length: 10 mm Diameter: 3.3 mm	Intensity: 40 mW/cm^2^ or 100 mW/cm^2^ (/SATA) Pulse frequency: 1 MHz or 3 MHz	20 min/day for 2 weeks	Clinical application of LIPUS for dental implants may promote osseointegration
Hsu et al. [31]	New Zealand rabbits Tibiae	Length: 8 mm Diameter: 3.6 mm Screw-shaped	Intensity: 50, 150, and 300 mW/cm^2^ (/SATA) Pulse frequency: 1 MHz	10 min/day for 30 days	LIPUS at 0.05–0.3 W/cm^2^ intensity may accelerate cell proliferation and promote the maturation of collagen fibers and support osteointegration
Liu et al. [32]	New Zealand rabbits Femur and tibiae	Length: 18 mm Diameter: 2.5 mm Screw-shaped	Intensity: 40 mW/cm^2^ (/SATA) Pulse frequency: 1.5 MHz	10 min twice a day (total 20 min) for 3 weeks	LIPUS has the potential to accelerate the osseointegration of dental implants
Zhou et al. [33]	Ovariectomized SD rats Tibiae	Length: 4.0 mm Diameter: 2.0 mm Pitch: 0.6 mm Screw-shaped	Intensity: 40 mW/cm^2^ (/SATA) Pulse frequency: 1.5 MHz	20 min/day for 2, 4, 6, 8, 10, and 12 weeks	LIPUS may enhance new bone formation, especially in an early stage, and improve osseointegration in osteoporotic bone as an auxiliary method
Ruppert et al. [34]	SD rats Femur	Length: 20 mm Diameter: 1.5 mm	Intensity: 30 mW/cm^2^ (/SATA) Pulse frequency: 1.5 MHz	20 min/day for 4 and 8 weeks, 5 days per week	LIPUS is superior to vibration for accelerating osseointegration and increasing bone–implant failure loads at 4 weeks
Jiang et al. [24]	*α*CGRP^+/+^ and *α*CGRP^−/−^ mice Maxillary first molar extraction sockets	Length: 1 mm Diameter: 0.6 mm Screw-shaped	Intensity: 30 mW/cm^2^ (/SATA) Pulse frequency: 1 MHz	20 min/day for 2 and 4 weeks	LIPUS can enhance osseointegration of dental implant by inducing local neuronal production of *α*CGRP, providing a new idea to promote peri-implant osseointegration and bone regeneration

CGRP: calcitonin gene-related peptide; LIPUS: low-intensity pulsed ultrasound; SATA: spatial average temporal average; SD: Sprague–Dawley.

## Data Availability

No data were used to support this study.
